# Recent advances in dysphagia management

**DOI:** 10.12688/f1000research.18900.1

**Published:** 2019-08-29

**Authors:** Joseph Triggs, John Pandolfino

**Affiliations:** 1 Department of Medicine, Division of Gastroenterology and Hepatology, Northwestern Medicine, Northwestern University, Feinberg School of Medicine, 676 St Clair Street, Suite 14-009, Chicago, IL, 60611-2951, USA

**Keywords:** dysphagia, manometry, FLIP, esophagus, EGJOO, jackhammer

## Abstract

The literal definition of dysphagia is “disturbed eating”. However, it is more accurately described in clinical practice as a sensation of food or liquid being stuck in the esophagus or chest. If this sensation is associated with pain, it is labeled odynophagia, and if it is associated with persistent obstruction and bolus retention, it is categorized as a food impaction. Through research and technological advances, we continue to expand our understanding of the etiologies and underlying pathophysiology relating to this complaint. However, for now, our clinical algorithms focus on endoscopy and manometry to break down dysphagia into three categories: obstructive dysphagia, esophageal motility disorders, and functional dysphagia. Here, we review some critical pitfalls in our current clinical diagnoses, new proposed underlying mechanisms of esophageal motor disorders, and developing technologies to aid in diagnosis and treatment.

## Introduction

Dysphagia is compartmentalized on the basis of location and whether or not there is evidence of a mechanical or inflammatory process leading to failed bolus transit. The initial distinction is focused on whether there is an oropharyngeal etiology or whether the abnormality is located below the upper esophageal sphincter. This distinction between oropharyngeal dysphagia and esophageal dysphagia can usually be gleaned from a careful history that focuses on the presence of immediate aspiration or cough with swallowing and other symptoms, such as nasopharyngeal regurgitation, voice changes, or the perception that there is an uncoordinated swallow at initiation. In esophageal dysphagia, localization of symptoms to the throat can often be misleading. Significant bolus retention and poor accommodation in the chest can be confused with an obstruction in the throat. This poor localization does not occur when patients localize the problem to the mid esophagus or lower chest ^1^. Performing an examination of cranial nerve function and watching the patient swallow water and eat a solid bolus can be extremely helpful. If oropharyngeal dysphagia is suspected, a video fluoroscopic swallow study performed by a speech pathologist should be obtained to localize the defect ^2^.

**Figure 1.**
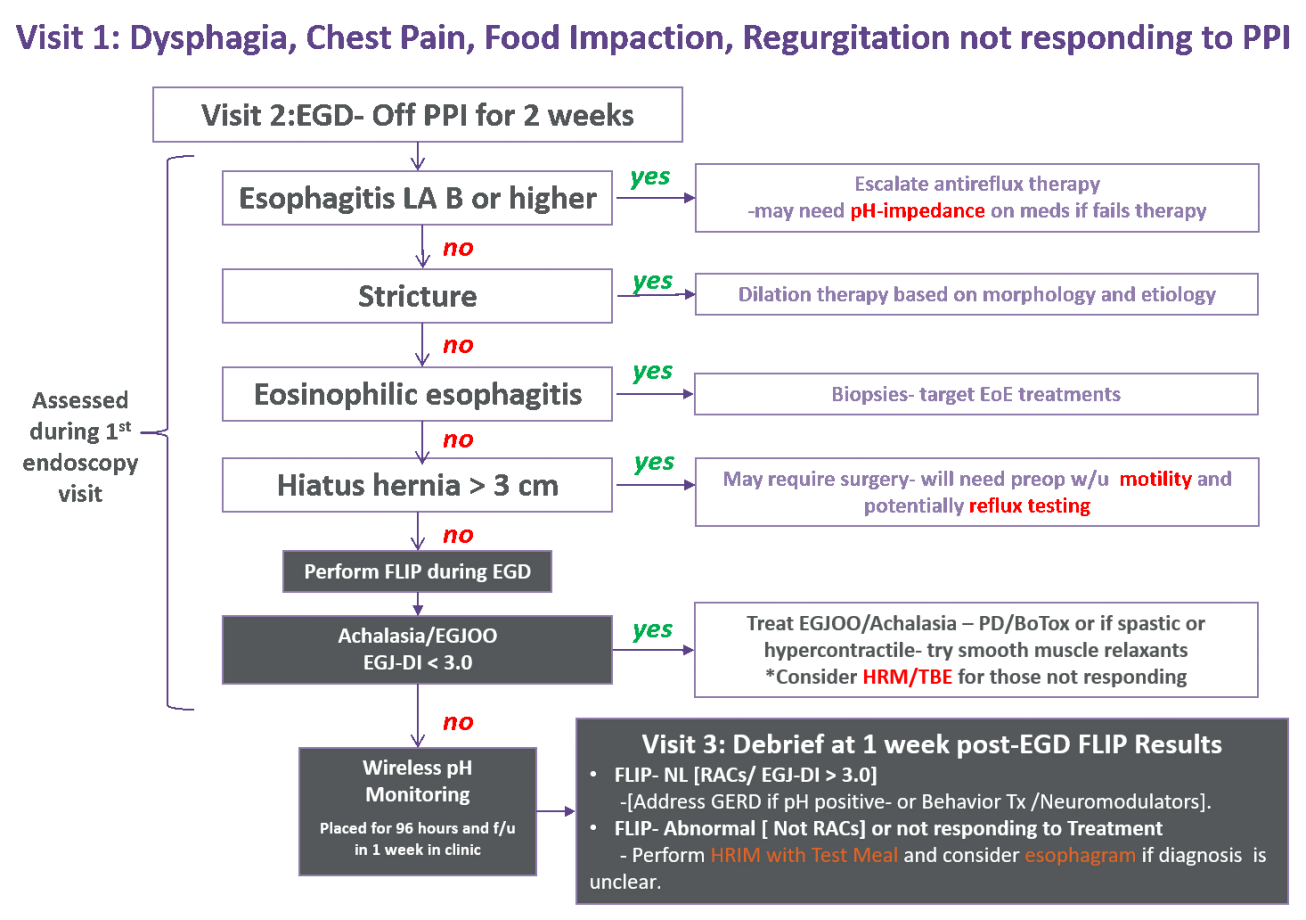
Treatment algorithm for patients presenting with esophageal dysphagia. EGD, esophageal dysphagia; EGJ-DI, esophagogastric junction-distensibility index; EGJOO, esophagogastric junction outflow obstruction; FLIP, functional luminal imaging probe; f/u, follow-up; GERD, gastroesophageal reflux disease; HRM, high-resolution manometry; LA, Los Angeles Classification; NL, normal; PD, pneumatic dilation; PPI, proton pump inhibitor; RAC, repetitive antegrade contraction; TBE, timed barium esophagogram; Tx, treatment. Figure courtesy of the Northwestern Esophageal Center.

Patients suspected of having esophageal dysphagia should be referred for an upper endoscopy as this test will help rule out mechanical obstruction or an inflammatory process or provide evidence that this may be an esophageal motor disorder. In fact, almost every algorithm focused on the management of esophageal symptoms begins with upper endoscopy as this will identify treatable etiologies and rule out malignancy. Although history can help assess the risk of malignancy and help distinguish a mechanical process from a motility disorder, there is no way around obtaining an endoscopy. Radiographic evaluation using various esophagram protocols can be helpful in assessing equivocal cases; however, using this approach as the initial test is not cost-effective and delays treatment as both positive and negative esophagrams necessitate endoscopic evaluation ^3^. Endoscopy provides an opportunity to treat strictures and obtain biopsies when the etiology is unknown or an inflammatory process is suspected. Patients without evidence of esophagitis (related to reflux, eosinophilic esophagitis (EoE), pill esophagitis, lichen planus, and so on), stricture/mass, or a large mechanical hiatus hernia should be referred for motility testing ( Figure 1). The standard algorithm is to refer patients for high-resolution manometry (HRM) to rule out a motility disorder that may explain the patient’s symptoms and direct therapy toward the motor abnormality driving the symptoms. This process has been focused on identifying achalasia and its subtypes so that directed therapy can be offered in a precision model ^4^. Patients without major motility abnormalities are often found to have functional dysphagia and are given neuromodulators and behavioral interventions ^5^.

Although management algorithms for dysphagia have not dramatically changed over the last decade, there have been major advances in diagnostic testing with the use of impedance technology and endoscopic interventions, and interesting observations have been made regarding the pathogenesis of motility disorders. Thus, the goal of this review will be to focus on these recent advances and discuss how they have improved our understanding of the disease process. A full description of each disease process that can present with dysphagia is beyond the scope of this review and instead this update will focus on some important pitfalls and concepts that are evolving within these diseases which may improve our management strategy.

## Diagnosis

The most important technology focused on assessing esophageal motor function in endoscopy-negative dysphagia is HRM. This technique continues to evolve since its introduction over a decade ago and there have been major changes in classification schemes and biomarkers of esophageal function during this time. The current classification scheme used to categorize esophageal motor dysfunction is the Chicago Classification (CC) ^6^. Although this scheme has had a major impact in improving manometric technique by reducing movement artifact and converting pressure tracings to a more intuitive pressure topography platform, there are limitations in using this classification scheme in clinical practice that, if not recognized, can lead to misdiagnosis and inappropriate interventions. The CC 3.0 focuses on describing patterns of contraction and pressurization and categorizes these on the basis of four specific components that promote normal antegrade emptying of the esophagus. These components mirrored what was originally conceptualized by using conventional manometry, but they are focused on providing a more accurate assessment of esophagogastric junction (EGJ) opening dynamics during swallowing, a better description of timing of peristalsis in terms of deglutitive inhibition, and more detail regarding propulsion of the bolus by a contractile pressure wave. The integrated relaxation pressure (IRP) provides a measure of the resistance forces to flow through the EGJ created by contact pressure when the lower esophageal sphincter (LES) is closed and the intrabolus pressure when the LES is open. The distal latency (DL) interval assesses whether deglutitive inhibition is intact by measuring the timing of smooth muscle contraction below the transition zone. Premature contractions that occur before the transition zone, or shortly thereafter, will lead to bolus compartmentalization (corkscrew or rosary bead esophagus) if the latency interval is shorter than 4.5 seconds. Propulsive function of the peristaltic wave is assessed by the ability of the esophagus to maintain a closed lumen in a seamless antegrade direction without gaps greater than 5 cm and with enough strength to maintain luminal closure. Strength of the contraction of the smooth muscle is measured by using the distal contractile integral (DCI), and the integrity of the wavefront is measured by using measures of peristaltic breaks below the 20 mm Hg isobaric contour. The CC was created by determining the upper limits of normal in asymptomatic controls for these new measures (IRP, DL, DCI, and peristaltic breaks) and applying this to large patient populations with dysphagia.

## Limitations of the Chicago Classification

Although the CC has advanced manometric technique, there are limitations with this approach that can have a negative impact on diagnosis of esophageal motor disorders. First, the classification scheme is highly dependent on the accuracy of the IRP and this metric can be fickle as it is dependent on position and the sensor technology. Thus, the diagnosis of an esophagogastric junction outflow obstruction (EGJOO) must be interpreted with caution and a decision regarding intervention should never be made on the basis of this measure alone. Additionally, the CC ignores the metadata of a manometry study as they are generated by using only 10 supine swallows based on the normative range data. Certainly, evidence of spastic or uncoordinated contractions between swallows or during provocative maneuvers should be considered in an interpretation of motor function. Last, abnormal motor patterns beyond what are seen in asymptomatic controls do not necessarily equate with a true primary motor disorder as anatomical issues, such as hiatus hernia and extrinsic compression, and subtle obstructions at the EGJ and esophageal wall can create motor patterns that mimic CC diagnoses.

### Esophagogastric junction outflow obstruction

EGJOO is a heterogeneous diagnostic group composed of patients with evolving achalasia, mechanical obstruction, or an artifact related to inherent issues with the IRP measurement. An elevated IRP can be a positional artifact or related to an extreme bend in the catheter, and the IRP may normalize with positional changes. Thus, it is extremely important to measure the IRP in both the supine and upright positions to assess whether this artifact is falsely leading to a diagnosis of EGJOO ^7^. There are complementary findings on HRM that increase the likelihood of a true obstruction, such as evidence of compartmentalized intrabolus pressurization, poor bolus transit, and concomitant hypercontractility. However, these findings have not been shown to be accurate enough to confirm an outflow obstruction, and other tests, such as a timed barium esophagram with a barium tablet or functional luminal imaging probe (FLIP) panometry assessment of EGJ opening, can be helpful in confirming a true obstruction. If a true obstruction is noted on these examinations, it is still difficult to determine whether this is achalasia in evolution, and further assessment may be required before achalasia treatment is considered in difficult and equivocal cases. Cross-sectional imaging or endoscopic ultrasound can be helpful but should be limited to EGJOO patients with dysphagia and overt obstruction noted on esophagram if a mechanical obstruction or pseudoachalasia presentation is suspected (older age at onset, weight loss out of proportion, and abnormalities at the EGJ during endoscopy).

### Jackhammer esophagus

Similar to EGJOO, jackhammer esophagus, which is diagnosed on the basis of two swallows with a DCI value above 8000 mm Hg*s*cm, is a very heterogeneous classification. Meeting these criteria, however, is not enough evidence to refer patients for invasive treatments. As mentioned above, this pattern can be associated with obstruction at the EGJ and is also seen in the context of gastroesophageal reflux disease (GERD) and EoE. Given this heterogeneity, further workup focused on ruling out an obstruction and empiric trials with smooth muscle relaxants should precede referral for myotomy ^8^. Many patients with diagnosed jackhammer esophagus will have a benign course, especially when this pattern is found incidentally during a pre-operative workup for GERD and thus observation and follow-up may also be reasonable. Currently, there are no criteria to distinguish true primary motor dysfunction in patients with jackhammer related to obstruction from features such as repetitive multipeaked contractions, caused by disrupted deglutitive inhibition, or long-duration post-peak contractions. Unfortunately, none of these patterns has sufficient predictive value to determine which patients will respond to therapy focused on reducing smooth muscle contractility. Recently, there has been substantial interest in the role of eosinophilic infiltration and inflammation of the circular muscle in primary motor disorders and this may lead to more aggressive assessment of muscle histology in the diagnostic paradigm ^9^.

## Advances in our knowledge of pathogenesis

### Eosinophilic myositis

EoE is an important etiology in dysphagia and currently is the leading cause of emergent food impaction in the US ^10^. This disorder is associated with a chronic immune/antigen-mediated eosinophilic inflammatory response that leads to fibrosis and remodeling of the esophageal wall ^11^. Although the cause of dysphagia in this patient group is believed to be related to mechanical narrowing of the lumen, there is evidence that EoE is associated with motor dysfunction ^12^. In fact, case reports documenting resolution of classic achalasia patterns after corticosteroid treatment in patients with diagnosed EoE have been published ^13^. There has also been emerging evidence that eosinophilia may extend beyond the mucosa and involve the smooth muscle of the esophagus in patients presenting with major motor disorders ^14^. Studies focusing on achalasia reported high rates of eosinophilic inflammatory ganglionitis in myotomy and esophagectomy specimens. Similarly, case series have described dense eosinophilic infiltration from biopsies in patients with jackhammer esophagus undergoing peroral endoscopic myotomy (POEM) ^15^. These studies have also reported the presence of eosinophil-derived secretory products with neurotoxic and cytotoxic effects within the circular smooth muscle, lending further credibility to the biologic plausibility of this pathogenic mechanism ^16, 17^. It is conceivable that these effects target and destroy the enteric neurons associated with normal peristalsis or that this inflammatory response leads to release of other factors that promote hypercontractility.

Although the above data only indirectly support this interesting hypothesis, more evidence is required before we begin to treat achalasia and jackhammer esophagus with steroid therapy. Future prospective studies should be performed to assess whether these abnormalities are truly pathogenic and not related to the underlying disease state.

### Opioid esophagus

The effects of opioids on gastric, small intestine, and colonic motility have been well described in the literature and most physicians understand that opioids can lead to constipation and reduce intestinal transit ^18^. Similarly, opioids can affect esophageal motility likely through similar mechanisms and this effect has been studied by using various pharmacologic intervention studies ^19^. In 1996, Penagini *et al*. reported on the effects of morphine and naloxone on esophageal motor function ^20^. Their findings suggested that the residual LES pressure during swallowing was increased and the duration of LES relaxation and percentage of relaxation were decreased by opioids. In addition, the authors found that peristaltic velocity was increased with minimal changes in contractile amplitude. These findings have been consistently found in other studies by using slightly different protocols in controls and thus it appears that opioids may alter the inhibitory component of esophageal peristalsis and LES relaxation. More recently, the Mayo team reported on the prevalence of CC diagnoses in 121 opioid users who were either on opioids at the time of manometry or off for at least 24 hours before the test ^21^. The authors found that the rates of both type III achalasia and EGJOO were more common in current users of opioids compared with those patients who had discontinued opioids for at least 24 hours.

These results suggest that opioid use can alter esophageal motility and can be associated with higher rates of major motor disorders in patients referred for esophageal manometry. Whether these effects will resolve with discontinuation of the opioid or normalize with administration of opioid antagonists is unclear. However, an understanding of these negative effects is important when evaluating patients with dysphagia on opioids.

## Evolving technologies

The incorporation of impedance technology to assess bolus transit and esophageal luminal diameter was a natural progression of motility assessment as the classification scheme of motor patterns was previously limited to an assessment of contractile and pressurization patterns. Additionally, impedance in its initial form was a simple dichotomous assessment of bolus transit that provided little discriminatory information in terms of objective and subjective outcomes. Subsequently, two new technologies that complement HRM have emerged: high-resolution impedance manometry and FLIP panometry.

### High-resolution impedance manometry

Although impedance assessment has been coupled to manometry for over 15 years, the impact of impedance on esophageal function testing in the context of dysphagia was minimal. It was not until impedance merged with HRM and was configured in an orientation that was similar to HRM that the impact of intraluminal impedance recordings was recognized. The early pioneers of this approach were from the lab of Taher Omari, and the initial work focused on assessing bolus passage dynamics across the pharyngo-esophageal segment as a non-radiologic assessment tool for aspiration in pediatric patients ^22^. This group advanced this approach into the adult population and the esophagus, creating the automated impedance manometry (AIM) platform, and they developed novel metrics that focused on the impedance signal as a marker of bolus distention and simultaneous intrabolus pressurization ^23^. They were able to show that these metrics could predict symptoms of dysphagia and also better discriminate symptomatic functional dysphagia patients and post-fundoplication patients ^24^. Other investigators have been modifying this approach to assess the inhibitory component of peristalsis and this approach will likely evolve further to provide volume flow estimates ^25^.

In parallel, the Northwestern group began exploring new techniques that provided a more quantitative aspect to the current combined impedance manometry algorithms by developing the esophageal impedance integral (EII) and the bolus flow time (BFT) ^26, 27^. EII was derived by developing a calculation of the cumulative impedance signal within the space–time domain of the swallow wave, focusing on the concept that drops in impedance are associated with volume-induced distention. Findings from this work suggest that EII correlates with fluoroscopy and symptom scores on the brief esophageal dysphagia questionnaire and this was superior to the standard HRM metrics used for CC ^28^. Using a similar approach and focusing on the EGJ, investigators conceptualized the BFT as a time measurement of EGJ opening by using impedance drops more than 90% of baseline as a marker of bolus presence and determining the time where a preferential flow gradient was present. Once again, this metric performed better than IRP or basal EGJ pressure in predicting bolus retention and symptoms ^28^. Newer approaches that attempt to use a more detailed assessment of the role of intraluminal impedance in predicting lumen geometry during swallowing are on the horizon.

### Functional luminal imaging probe panometry

**Figure 2.**
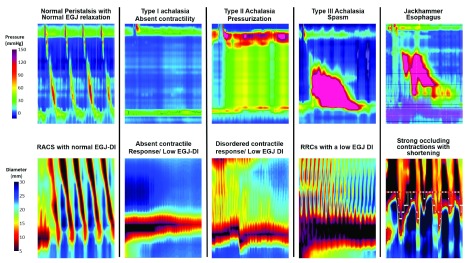
High-resolution manometric patterns (top) and the corresponding functional luminal imaging probe panometry patterns (bottom). EGJ, esophagogastric junction; EGJ-DI, esophagogastric junction-distensibility index; RACS, repetitive antegrade contractions; RRC, repetitive retrograde contraction.

FLIP panometry is an adaptation of impedance planimetry that uses sensors that are configured in a high-resolution orientation to provide a three-dimensional image of the esophageal lumen ^29^. By combining data on lumen dimensions and adding a pressure sensor, mechanical properties of the esophagus, such as distensibility and compliance, can be measured by assessing diameter/volume pressure changes. This technique is performed while the patient is sedated during standard upper endoscopy. The approach focuses on assessing distensibility of the EGJ and has been shown to provide useful complementary information regarding EGJ function in achalasia and post-surgical obstruction ^30, 31^. More recently, this technology was adapted to assess esophageal motor function in response to a sustained volumetric distention ^32^. The volumetric response will elicit secondary distention-mediated contractile activity and this can be visualized by converting diameter measurements into a color topography plot that illustrates diameter changes over a space–time domain. The reductions in diameter represent contractions and these contractions can be assessed on the basis of their direction and ability to occlude the lumen. Antegrade contractions occurring in a repetitive sequence spaced about 6 to 8 seconds apart are considered to be a normal response to sustained volumetric distention and this pattern is associated with normal peristalsis on HRM ( Figure 2). Contractions can occur in a retrograde direction and this pattern has been seen in the context of spastic disorders, EGJ obstruction, and chronic opioids. Failure to elicit contractions is associated with aperistalsis and weak peristalsis and may represent a myogenic dysfunction related to dilatation and atrophy or a neurogenic dysfunction related to impaired triggering. When these patterns and an already-validated methodology to assess EGJ opening are used, the patterns of response can be conceptualized into a motility classification scheme similar to the CC ( Figure 2) ^33^. Although this device is currently being used as a complementary tool in the management of dysphagia, the unique advantages of providing both motility and biomechanical measures while the patient is sedated could make it a more mainstream diagnostic tool for the management of esophageal diseases in the future.

## Interventions

### Peroral esophageal myotomy

Over the past decade, submucosal endoscopy has developed as a new tool in the armamentarium of the therapeutic endoscopist. Via these techniques, POEM has quickly been adopted as one of the three principal treatments for achalasia, along with pneumatic dilation and laparoscopic Heller myotomy ^34, 35^. POEM was introduced in an animal model in 2007, when Pasricha *et al*. described creating a submucosal tunnel using a biliary dilating balloon followed by circular muscle myotomy using a needle knife ^36^. In 2008, Inoue *et al*. performed the first POEM in humans and published a case series of 17 patients for the treatment of achalasia ^37^. POEM was first performed for uncomplicated achalasia, but there is currently no consensus regarding formal indications, and substantial work has been carried out to study various treatment applications, including achalasia (all three clinical subtypes) and non-achalasia motility disorders (distal esophageal spasm, EGJOO, and jackhammer esophagus) and following failed prior LES targeted therapy for achalasia ^38^. This technique is continuing to mature but outcome data are promising; more than 90% of patients have reported clinical improvement ^39, 40^.

## Summary

Our understanding of the pathophysiology and underlying mechanisms driving dysphagia continues to evolve along with the technologies we use to assess and make clinical decisions for our patients. However, for now, we continue to rely on upfront endoscopy to rule out mechanical obstruction and malignancy and HRM for the diagnosis of esophageal motor disorders in endoscopic-negative dysphagia. In the near future, new technologies like FLIP will bring motility testing into the index endoscopy and have the potential to expedite care and improve our ability to phenotype patients. HRM is also evolving and will aid in the adjudication of difficult borderline cases and spastic disorders. Furthermore, endoscopic ultrasound and the use of endoscopic mucosal dissection techniques to obtain deep muscle biopsies may assume a more prominent role if eosinophils are truly involved in esophageal dysmotility beyond EoE. Thus, management of dysphagia is evolving quickly in parallel with new technologies.
